# Growth front smoothing effects in extremely high pressure vapor deposition

**DOI:** 10.1038/s41598-020-69269-4

**Published:** 2020-07-23

**Authors:** Nicholas Pittman, Toh-Ming Lu

**Affiliations:** Department of Physics, Applied Physics and Astronomy, Rensselaer Polytechnic Institute, Troy, NY USA

**Keywords:** Computational methods, Structural properties

## Abstract

Recent experimental chemical vapor depositions of silicon at extreme pressures of ~ 50 MPa (~ 500 atm) have been observed to generate remarkably smooth surfaces not predicted by low-pressure deposition models. In this paper, we propose an anti-shadowing mechanism where the collision of particles within the valleys of the surface growth front leads to smoothening. We conduct Monte Carlo simulations to simulate the evolution of film roughness at pressures between 1 and 50 MPa. We observe that surface roughness approaches an asymptotic invariant value that follows power law behavior as a function of pressure. The film thickness at which invariance begins is shown to have a similar power law behavior with respect to pressure. Our simulated results compare favorably with recent experimental observations and provide insight into the fundamental mechanisms underlying film evolution at pressures between one and hundreds of atmospheres.

## Introduction

The morphology of thin films has long been a subject of great interest to researchers, particularly to those working with the technological applications of thin film deposition, as their morphology can directly affect the mechanical, chemical, electrical, optical, and magnetic properties of the films produced. Physical vapor deposition (PVD) and chemical vapor deposition (CVD) are two of the most common vapor deposition techniques^1,2^. Film growth can occur under far-from-equilibrium conditions, meaning that conventional statistical mechanics centered around equilibrium cannot be applied to predict the evolution of the surface during the deposition^3,4^. For instance, attempting to obtain thermodynamic quantities from the partition function is not feasible, so we must resort to simulation techniques such as Monte Carlo methods. These techniques mimic film growth in a computerized simulation by simulating particles being deposited from a vapor phase onto a developing surface. The aggregation of atoms on the surface forms the growth front.

One surface statistic used to describe the vertical roughness of the surface is called the root-mean-square roughness, or RMS, defined as $\omega (t)=\sqrt{<({h}^{2}\left(r, t\right)-{\overline{h}}^{2}(t))>}$, where $h$ is the height of the surface at position *r* and $\overline{h}$ is the average height of the surface^3,5,6^. This parameter is essentially the standard deviation of vertical heights, meaning that a high RMS indicates a rougher surface with large vertical fissures.

The characteristics of the surface growth depend on many parameters, including the angular distribution of the incident particles, the pressure of the deposition, and how atoms diffuse (if at all) once they reach the surface, among many other factors^7–10^. As one increases the deposition pressure, the mean-free-path (MFP) of the depositing vapor decreases, meaning that more and more collisions are occurring between depositing molecules in the vapor phase. These collisions alter the angles at which the incident particles reach the surface; as pressure increases, the collisions tend to spread out the angular distribution of the incoming particles before striking the surface^11^. Although the low pressures of PVD correspond to MFPs on the order of millimeters or larger^1^, CVD can achieve much greater pressures and smaller MFPs^2^.

In order to achieve higher deposition rates without an undesirable increase in temperature, it is desirable to push the deposition pressure beyond one atmosphere. However, this high pressure usually induces the formation of undesirable clumps of particulates that eventually deposit onto the surface^12^. To avoid the undesirable gas phase reactions and particulate formation that occur in this regime, a strategy has recently been developed to perform extremely high pressure CVD depositions in between a series of plates separated by very small distances on the order of 10 μm^12^. This has been shown to reduce/eliminate the presence of aggregates on the surface in experimental depositions of up to 33 MPa (~ 326 atm), with even greater pressures of up to 50 MPa (~ 500 atm) theorized as possible. It is believed that by making the interlayer spacing so tight, there is little chance one particle will undergo sufficiently many collisions to form an aggregate^12^. As a result, this new deposition chamber geometry has opened the door for extreme pressure depositions that would have previously caused excessive particulate formation.

The films obtained in these setups are extremely smooth, even though this is at odds with conventional understandings of growth front morphology formation under high pressure. According to the shadowing model (explained below), as the pressure increases, more atoms are obliquely incident (i.e., have trajectories close to parallel to the surface), causing severe shadowing and giving rise to significant RMS roughness.

The shadowing effect may be understood rather intuitively. The core concept of shadowing (which will also eventually lead to anti-shadowing) is that during the course of a film deposition, small bumps or hills naturally form on the surface under non-equilibrium conditions, simply due to random variations in the number of particles that have deposited in certain areas. Initially, before the formation of these bumps, there is no “obstruction” of any areas of the surface by the rest of the growth front. Once the hills have formed, however, any particle with a trajectory not normal to the surface (more severe if it has a large angle with respect to the surface normal) will suddenly not be able to reach the “shadows” cast by these hills. This difference in accessibilities between areas on the surface leads to the hills growing larger as more particles impact them, while the areas at the base of the hills receive proportionally fewer particles. As such, the shadowing model attempts to explain the *growth of hills on the surface as a consequence of “shadows” cast by hills on the surface (i.e., a difference in the particle flux on hills and in valleys between them because of surface features obstructing certain areas).* Quantitatively, for film growth under shadowing, the RMS roughness grows as a function of time $t$ in the form $\omega \sim {t}^{\beta }$, where $\beta =1$ is the universal value observed in the presence of shadowing^13,14^. As such, in shadowing models the RMS grows linearly and indefinitely as the simulation progresses.

**Figure 1:**
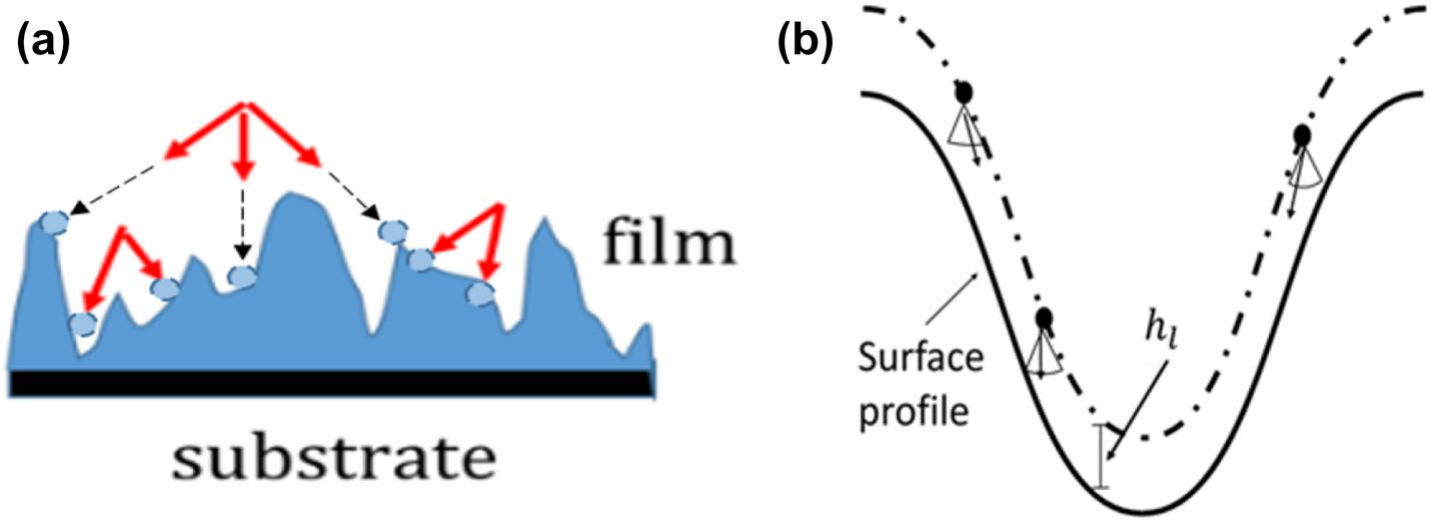
(**a**) A high pressure deposition with a mean-free-path smaller than the surface roughness feature size. The gas phase collisions can occur below the highest points in the rough surface, as shown by the arrows on the lower left. The circular outlines on the surface represent where each of the particles with velocities given by the red arrows would deposit (note that in the actual solid-on-solid simulation model, after initial impact these particles would also fall straight downwards until they land on top of a previously deposited particle). (**b**) Gas phase collisions occur within a valley of the surface profile where the mean free path ${h}_{l}$ is smaller than the height of the valley. The incident particle is initialized at a height ${h}_{l}$ above the local surface.

In this paper, we perform Monte Carlo simulations to investigate the growth mechanisms that determine the surface morphological development at these extreme pressures. We focus our attention on the evolution of the RMS roughness for pressures between 1 and 50 MPa. Our model is based on an “anti-shadowing” effect^15^ that occurs when the MFP of depositing particles is comparable to the size of fissures on the growth front. In this pressure regime, collisions can occur inside of the valleys on the surface (Fig. 1a), halting the roughening of the surface. This anti-shadowing mechanism has been suggested to explain the relatively smooth surface observed under CVD at atmospheric pressures^16,17^.

To extend the earlier analogy to the anti-shadowing model, we may loosely say that the origin of the invariant roughness phenomenon is due to the tendency of a particle’s last collision to be close to the surface (in a valley), leading to more opportunities for previously shadowed areas to receive particles. That is, *the cessation of roughness growth (invariant roughening stage of growth) is proposed to arise as a consequence of a difference in the particle flux received by the top of hills and the valleys once collisions are presumed to occur below the max surface height.*

Merkh et al.’s paper was an early implementation of the concept of an anti-shadowing mechanism in a Monte-Carlo simulation and demonstrated the concept in a deposition by manually varying the *h*_*l*_ of a simulation with a constant cosine angular flux pattern^15^. Few connections to pressure were made, however, since at that time experimental data on the growth front characteristics for depositions at hundreds of atmospheres was not available. Although the difficulties the authors cite in determining the mean free path-pressure relationship still exist, we now have a motivation to use an approximation of the relationship to attempt to explain these new experimental observations. Our current work quantitatively predicts the growth front characteristics as a function of pressure to hundreds of atmospheres, which is immediately relevant to experimental observations. By analyzing the invariant RMS versus pressure curve, we discovered the scaling behavior (power law dependence of invariant roughness on pressure) which was not known previously, and along the way come across new developments relating to the angular distribution of incident particles.

CVD under confined architectures and extreme pressures of these sorts may open the door to significantly increased deposition speeds in thin films across a wide range of applications. The increased deposition rate associated with these pressures assists in rapid production, and the smoothness of the resulting films may be key in producing the high-quality films needed to make low-cost hydrogenated amorphous silicon solar cells^12^. In addition, extreme pressure depositions are one proposed method of applying a conformal coating to the interiors of nano-architectures, which would prove very advantageous to the fabrication of complex nanostructures^18^.

## Methods

Our simulation uses a lattice to model the space inside a vapor deposition setup, and then evolves particles downwards onto a substrate where they accumulate to form the film. A detailed overview of the simulation process has been published elsewhere^11^. In brief, for each given particle within a single step in the simulation, the particle is created at a random spatial point (x, y) and some height z in the lattice, and with an initial velocity direction. The direction is determined by sampling from an angular probability distribution based on approximating the effects of binary hard-sphere collisions between depositing particles^11^. This distribution is pressure-dependent, meaning that based on the pressure of the deposition, the flux distribution will vary^11^.

The total lattice size in the (x,y) plane is 512 units by 512 units. The crucial change to previous simulations that we introduce involves the method of determining what z position that the particle is initialized at. In our simulation, this value is the local height of the surface plus the MFP of the vapor (which is a function of the pressure^19^), since the angular flux distribution is calculated based on the expected trajectories of particles at the point of their last expected collision before surface impact. Some particles may have their last gas phase collision very close to the surface, while others will have their last collision far above the surface. On average, though, the last collision occurs about 1 MFP above the surface. For sufficiently low pressures, the MFP is larger than the maximum surface height, ${h}_{max}$, so we can choose to initialize particles only one lattice unit above the maximum surface height, at ${z= h}_{max}$+1. Progressing the simulation in this manner saves the time of creating all our particles far above the surface and having to evolve them downward, considering individual collisions and changing trajectories. In previous simulations of low pressure depositions, this step was always able justifiable, as the mean free paths were of a length scale the surface RMS roughness would never reach.

For high pressures on the order of atmospheres, however, as the simulation progresses and the surface features grow in depth, the size of the MFP will be comparable to or exceeded by the size of the surface features. Now, it is possible for particles to be initialized (i.e., make their final collision before surface impact) inside the valleys on the surface. This is shown in Fig. 1b, in which the MFP is denoted by the simulation parameter *h*_*l*_. The algorithms of many simulations used to model low pressure depositions *fail* to incorporate this behavior as the pressure is increased towards the atmospheric regime, since they initialize all particles one unit above the max surface height^11^. A key innovation of our code is that it contains a clause that compares the maximum possible size of valleys on the surface (surface maximum height minus surface minimum height) to the MFP at each step. The default protocol in our simulation is to use *h*_*l*_ as the mean free path*,* and only when the MFP is larger than the maximum valley size are particles generated at one unit above the maximum height to increase the speed of the simulation^11^.

It has been noted in the literature, however, that in experimental vapor depositions at high pressures, it may be difficult to determine the physical mean free path as a function of pressure. This difficulty arises due to local variations in the concentration (and thereby pressure) of the gas phase, which is affected by the physical shape of the deposition chamber as well as the flow rate of the inlet^15^. With this limitation in mind, we will determine an approximate mean free path using the standard binary hard sphere collision formula^19^. In order to apply this formula to determine the MFP, we must decide on a value of chamber temperature and particle diameter. We set our chamber temperature to approximately room temperature at 300 K, in line with previous work^11,15^, which will also allow us to make some later comparisons between our results and those in Ref. 15. Due to the elevated substrate temperature in the experimental depositions, it is likely that the true chamber temperature is between room temperature and the temperature of the substrate, so our MFPs may be underestimates of the true MFP of particles at a given pressure in this regard. Note that although the detailed surface energetics and temperature will affect the adatom surface mobility upon arrival at the surface and the time to reach the invariant roughness, it will not affect the scaling behavior of the final invariant roughness as a function of pressure, which will be displayed later in the text in Fig. 3b. That is, the exponent of the invariant roughness vs. pressure power law remains unchanged.

In deciding on a particle diameter, we make another approximation: within a typical CVD of a silicon film, in addition to the gaseous silane precursor molecules, a carrier gas such as helium is used within the chamber. In our simulations, we will use the diameter of a silicon atom of $2.12 \AA$ to calculate the approximate MFP in the chamber. We choose this representative particle diameter since a particle of Si is smaller than SiH_4_, but larger than He. We must keep these approximations in mind when connecting our results to pressure. Finally, we note that this diameter of silicon will also fix the physical length corresponding to one lattice unit in the simulation to $2.12 \AA$.

Once the particle is detected to have impacted the growth front, it is deposited. In the solid-on-solid model that we employ, it will fall downwards (in the ballistic model, it would stick). It has been previously observed that the effects of this choice on roughening is rather negligible compared to other factors^15^. Diffusion is allowed on the surface in our model, mirroring the value for silicon previously used successfully in the literature^15^.

Here we are dealing with amorphous silicon films whose morphologies are determined by the deposition spray patterns rather than any crystalline structure. The experimental deposition with which we compared our results used a sufficiently low substrate temperature of 613 K with an extremely high deposition rate so that crystallization did not occur^12^.

## Results and discussion

**Figure 2:**
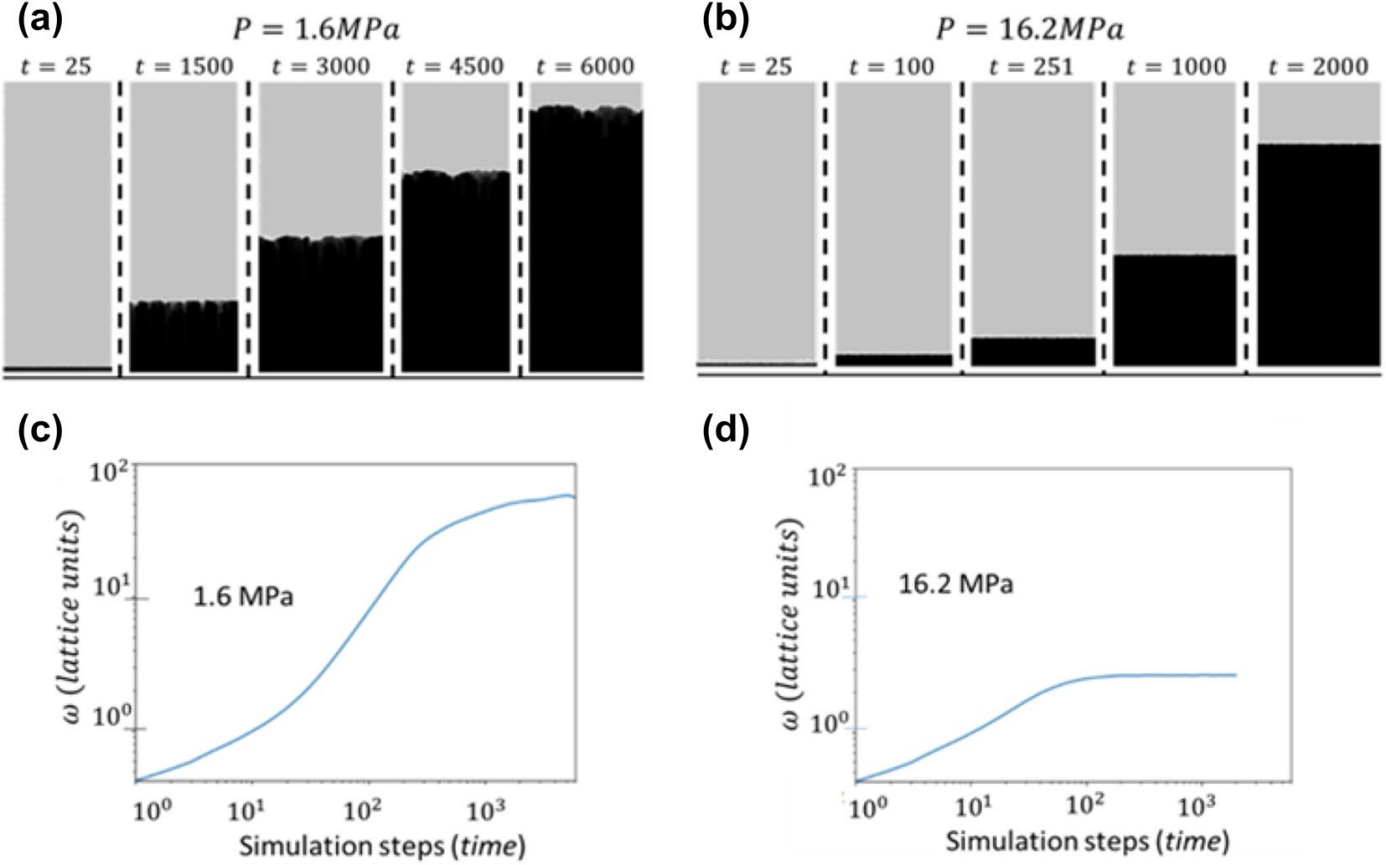
Side views of the surfaces grown at (**a**) 1.6 MPa and (**b**) 16.2 MPa at various time steps during the simulation. For each pressure, the first two columns are images before roughness invariance begins, the third is taken at the approximate time of transition to roughness invariance, and the last two are taken after invariance has been achieved. Note how the higher pressure surface achieves invariant roughness in a shorter time span, and stabilizes at a smoother morphology than the lower pressure. Sub-plots (**c**,**d**) are graphs of surface RMS roughness as a function of time for deposition pressures of 1.6 MPa and 16.2 MPa, respectively.

Figure 2a,b show the time evolution of a cross section view of the surface growth front simulated with a pressure of 1.6 MPa (16 atm, MFP ~ 19 lattice units) and 16.2 MPa (160 atm, MFP ~ 2.7 lattice units). In both cases, the starting surface is flat. As time evolves, the surface RMS $\omega$ increases. Eventually, the RMS stops growing and stabilizes, but this happens earlier for the higher pressure. Figure 2c,d show the simulated $\omega$ versus time curves for deposition pressures of 1.6 MPa and 16.2 MPa, respectively. This shape is typical of a deposition at any pressure within the regime we are investigating. Initially, the $\omega$ value increases. But as the simulation progresses, the $\omega$ growth begins to deviate from this pattern, slowing until it approaches a constant value of ${\omega }_{inv}$, the invariant roughness. Physically, this means the surface is growing rougher (increasing standard deviation of surface heights) as time progresses, but after a certain amount of time, the surface reaches a peak roughness. After this critical time, the surface does not become any rougher, even as more particles are deposited. Also, as one can see, the $\omega$ for the 16.2 MPa case levels off very early compared to that of 1.6 MPa deposition. The value of $\omega$ stabilizes at a value of less than 10 lattice units in the 16.2 MPa case, compared to about 180 units for the 1.6 MPa case.

**Figure 3:**
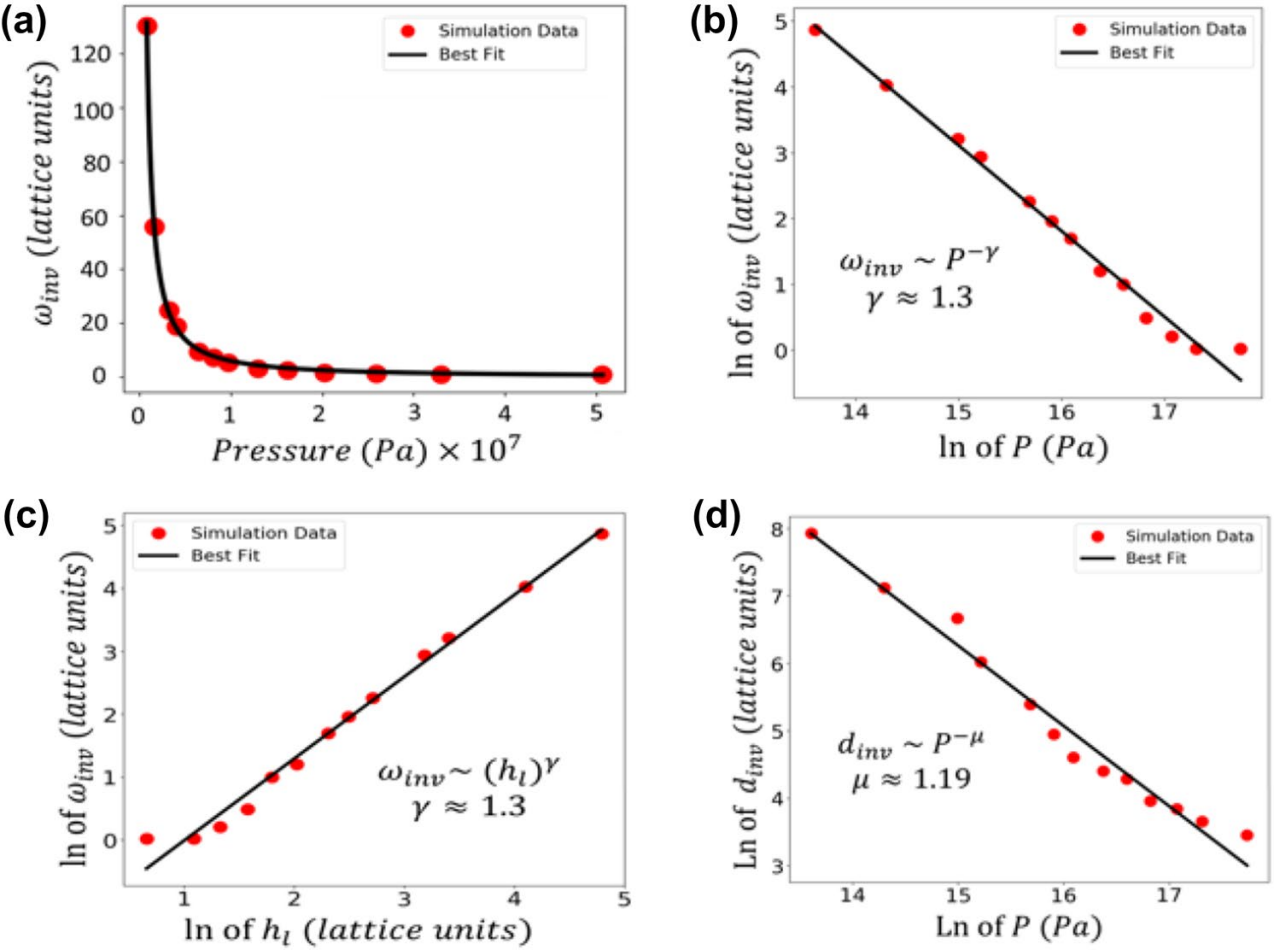
Invariant RMS ${\omega }_{inv}$ as a function of pressure on (**a**) linear axes and (**b**) logarithmic axes. The slope on the log–log plot is $-1.30\pm 0.04$ and the y-intercept is $22.6\pm 0.7$. (**c**) Invariant roughness ${\omega }_{inv}$ as a function of *h*_*l*_ on logarithmic axes. The slope is $+1.30\pm 0.04$ and the y-intercept is $-1.3\pm 0.1$. (**d**) Log–log plot of the invariant thickness ${d}_{inv}$ as a function of pressure. The slope is $1.19\pm 0.06$ and the y-intercept is $24.1\pm 0.9$. In all of the log–log plots, the slope of the line of best fit (or its magnitude) is the exponent in the power law relationship between the two variables, and the y-intercept is the natural logarithm of the coefficient of the power law (in lattice units).

To compute the invariant roughness ${\omega }_{inv}$, the average of the last 10 data points in the roughness versus time graphs was taken and recorded as the invariant roughness (after checking that the graphs leveled off correctly). These ${\omega }_{inv}$ are plotted as a function of pressure in Fig. 3a on a linear plot and in Fig. 3b on a log–log plot. The data very clearly form a strong linear relationship on the log–log plot, indicative of power-law behavior,${\omega }_{inv}\sim {P}^{-\gamma }$, where *P* is the pressure. From the orthogonal distance regression (ODR) line of best fit, we determine the linear fit on the log–log plot to have a slope and standard error of $-1.30\pm 0.04$, which describes an invariant roughness exponent of $\gamma =1.30\pm 0.04$. The line of best fit has a y-intercept of $22.6\pm 0.7$, which is the natural log of the coefficient of the power law in lattice units. In all of our cases, to recover the equation of the curve of best fit on linear axes, one would write ${\omega }_{inv}\sim {P}^{-\gamma }$, with the constant of proportionality determined by raising $e$ to the y-intercept. The resulting ${\omega }_{inv}$ values are in lattice units.

Based on the power law relationship between ${\omega }_{inv}$ and pressure, we expect to see a clear trend in experimental films grown at different pressures. According to our data, depositions with very high deposition pressures should produce very smooth films. We do indeed see this correlation in an experimental result^12^, in quite good agreement with our simulation’s predictions. He et al. outlines an experimental silicon deposition at a pressure of 33 MPa using the new deposition geometry described in the introduction. Operating at a substrate temperature of 613 K, the authors produced a surface whose final RMS was 4 angstroms. In comparison, our simulated result for a deposition at 33 MPa yielded an invariant RMS of ~ 1 lattice unit, corresponding to a physical roughness of ~ 2 angstroms. This is considered to be close to the experimental finding, considering the various approximations we have made with regard to the chamber temperature, particle diameter, and homogeneous pressure (as discussed in detail in the introduction).

By plotting the invariant roughness versus *h*_*l*_ on logarithmic axes, we may make some additional conclusions. From the slope of the line in Fig. 3c, we obtain a growth exponent of $1.30\pm 0.04$ for the power law relating ${\omega }_{inv}$ to *h*_*l*_; that is, ${\omega }_{inv}\sim {({h}_{l})}^{1.30}.$ By comparing our ${\omega }_{inv}$ vs. *h*_*l*_ plot with the similar figure in Merkh et al., we can determine the effects of the angular distribution on the behavior of ${\omega }_{inv}$. In the work of Merkh et al*.*, a fixed cosine flux distribution was employed at all *h*_*l*_ values, whereas we used a binary collision distribution. Besides the angular distribution, all other simulation factors such as diffusion parameters and chamber temperature were identical (except for Merkh et al*.*’s initial substrate pattern, which would not affect the ${\omega }_{inv}$ value, as discussed below).

Comparison between the two graphs reveals that our ${\omega }_{inv}$ value at each particular *h*_*l*_ was about 1.5–2.5 times as large as Merkh et al*.*’s. For instance, for an *h*_*l*_ of about 25 lattice units Merkh et al*.* obtained an ${\omega }_{inv}$ value of around 10 lattice units, while for the same *h*_*l*_ we obtained an ${\omega }_{inv}$ of about 25 lattice units. An inspection of Merkh et al*.*’s figure shows that this trend of our value being about twice as great is *approximately* uniform across the *h*_*l*_ values pictured. This would imply that the power law exponent relating ${\omega }_{inv}$ to *h*_*l*_ is not affected by the choice of angular distribution.

To summarize the above, *although* using a different angular distribution affects the numerical value of ${\omega }_{inv}$ corresponding to a particular *h*_*l*_, it will *not* affect the scaling behavior of ${\omega }_{inv}$ as the *h*_*l*_ is changed. That is, for any family of simulations using a particular angular distribution, the ${\omega }_{inv}\sim {({h}_{l})}^{1.30}$ relationship will be true, independent of the angular distribution used.

One may wonder what the effects of starting with a surface that had some initial roughness might be, as was done in Merkh et al. For a starting roughness less than the expected invariant roughness at that pressure, we may simply identify a later step in our deposition (say, *t* = 500 steps) with the “beginning” of this other simulation, and as such expect the surface to tend towards the same invariant RMS value. In Merkh et al.’s simulations with non-flat initial surfaces, in no case was an initial roughness higher than an observed invariant roughness, so we were able to assert that the initial pattern did not affect the ${\omega }_{inv}$ value. More interesting is the question of a starting surface that is rougher than the invariant roughness. Since our depositions never reach this roughness, we cannot make the above identification with a later step in the same deposition. We conjecture that the surface will likely start becoming less rough and approach the same invariant roughness predicted by our flat-substrate data for the given pressure, but we did not investigate this in our work.

Finally, in realistic film growths, one is not only interested how smooth the film would ultimately become at a particular deposition pressure, but also at what thickness the film being grown will reach that predictable smoothness/morphology. This thickness ${d}_{inv}$ can be estimated by multiplying the approximate step at which the invariance transition occurs by the number of particles deposited per step (to arrive at total deposited particles at this time), and then dividing by the area of the lattice. This yields the average height of the surface in terms of lattice units, which can then be multiplied by particle diameter to estimate the thickness at the beginning of invariance. Figure 3d displays the relationship between ${d}_{inv}$ and pressure. A linear trend on the log–log plot again indicates a power law form for the data, ${d}_{inv}\sim {P}^{-\mu }$, with best fit on the log–log plot indicating an exponent of $\mu =1.19\pm 0.06$ and a y-intercept of $24.1\pm 0.9$. The relationship displayed in Fig. 3d could prove useful for optimizing the mass fabrication of films that have just reached their (predictable) invariant roughness/smoothness level, which may be applicable in solar cell manufacturing that requires such mass production of uniform wafers. In addition, as discussed in the final paragraph of the introduction, this relationship may be useful in the uniform mass coating and construction of nanostructures with a desired surface smoothness^18^.

## Conclusion

In this paper we have illustrated some key effects that occur at the growth front of an extreme pressure CVD deposition. Most generally, we observed that the RMS surface roughness will stabilize (invariant roughness) after some time under very high pressure deposition. A power law relationship is obtained between the invariant roughness and the pressure applied in the deposition, ${\omega }_{inv}\sim {P}^{-\gamma }$ with $\gamma =1.30\pm 0.04$. Higher deposition pressures and the associated smaller MFP lead to smoother surfaces (smaller invariant roughness) that stabilize earlier than lower pressures. It is further determined that the overall choice of angular distribution affects the ${\omega }_{inv}$ of a simulation, even at extremely high pressures. However, it is argued that the growth exponent relating ${\omega }_{inv}$ to *h*_*l*_ is independent of the angular distribution used. The invariant thickness of the films when invariant roughness occurs is also estimated as a function of pressure and is also in the form of a power law, ${d}_{inv}\sim {P}^{-\mu }$, with $\mu =1.19\pm 0.06$. Our simulation’s agreement with experiment supports the idea that an anti-shadowing mechanism may account for observed behavior of film roughness at very high pressures. These results may serve as a guide to experimentalists who are interested in the morphological evolution of films grown under extremely high pressures, in particular the surface roughness and mechanisms that underlie it.

## Data Availability

The code used to generate this data, and the specific simulation data analyzed to reach these results, is available from the authors upon reasonable request due to very large file sizes.
